# A global checklist of the Bombycoidea (Insecta: Lepidoptera)

**DOI:** 10.3897/BDJ.6.e22236

**Published:** 2018-02-12

**Authors:** Ian J Kitching, Rodolphe Rougerie, Andreas Zwick, Chris A Hamilton, Ryan A St Laurent, Stefan Naumann, Liliana Ballesteros Mejia, Akito Y Kawahara

**Affiliations:** 1 Natural History Museum, London, United Kingdom; 2 Muséum national d’Histoire naturelle, Sorbonne Université, Institut de Systématique, Evolution, Biodiversité (ISYEB), UMR 7205 – CNRS, MNHN, UPMC, EPHE, Paris, France; 3 CSIRO - Australian National Insect Collection, Canberra, Australia; 4 Florida Museum of Natural History, University of Florida, Gainesville, United States of America; 5 Hochkirchstrasse 71, Berlin, Germany; 6 CESAB, Centre de Synthèse et d'Analyse sur la Biodiversité, Aix-en-Provence, France

**Keywords:** Anthelidae, Apatelodidae, Bombycidae, Bombycoidea, Brahmaeidae, Carthaeidae, Classification, Endromidae, Eupterotidae, Phiditiidae, Saturniidae, Sphingidae

## Abstract

**Background:**

Bombycoidea is an ecologically diverse and speciose superfamily of Lepidoptera. The superfamily includes many model organisms, but the taxonomy and classification of the superfamily has remained largely in disarray. Here we present a global checklist of Bombycoidea. Following Zwick (2008) and Zwick et al. (2011), ten families are recognized: Anthelidae, Apatelodidae, Bombycidae, Brahmaeidae, Carthaeidae, Endromidae, Eupterotidae, Phiditiidae, Saturniidae and Sphingidae. The former families Lemoniidae and Mirinidae are included within Brahmaeidae and Endromidae respectively. The former bombycid subfamilies Oberthueriinae and Prismostictinae are also treated as synonyms of Endromidae, and the former bombycine subfamilies Apatelodinae and Phitditiinae are treated as families.

**New information:**

This checklist represents the first effort to synthesize the current taxonomic treatment of the entire superfamily. It includes 12,159 names and references to their authors, and it accounts for the recent burst in species and subspecies descriptions within family Saturniidae (ca. 1,500 within the past 10 years) and to a lesser extent in Sphingidae (ca. 250 species over the same period). The changes to the higher classification of Saturniidae proposed by Nässig et al. (2015) are rejected as premature and unnecessary. The new tribes, subtribes and genera described by Cooper (2002) are here treated as junior synonyms. We also present a new higher classification of Sphingidae, based on Kawahara et al. (2009), Barber and Kawahara (2013) and a more recent phylogenomic study by Breinholt et al. (2017), as well as a reviewed genus and species level classification, as documented by Kitching (2018).

## Introduction

Bombycoidea is one of the most charismatic and well-studied moth lineages. The superfamily is mosty diversified in the intertropical region of the globe and currently includes ten families and more than 500 genera (van Nieukerken et al. 2011). Bombycoidea includes many model organisms (e.g., *Bombyx mori* Linnaeus, 1758, *Manduca sexta* Linnaeus, 1763, *Hyalophora cecropia* Linnaeus, 1758) that serve pivotal roles in studies on genetics, physiology, and development (see Roe et al. 2009). They are also economically important (e.g., pests, sericulture) and are frequently used as educational tools due to their large body size, attractiveness and ease of rearing in captivity (e.g., atlas moth, *Attacus atlas* (Linnaeus, 1758) and luna moth, *Actias luna* (Linnaeus, 1758)). Despite their central role in science and outreach, a comprehensive, vetted global checklist of Bombycoidea taxa is lacking, and the taxonomy of the group has been unstable. Existing taxonomic lists have focused on particular groups (e.g. Sphingidae, Kitching and Cadiou 2000), or faunas (e.g. Neotropical Bombycoidea, various authors in Heppner (1996)), but a comprehensive update of the entire superfamily is much needed. The morphology-based phylogenetic studies of Minet (1991) and Minet (1994) were seminal for the modern classification of Bombycoidea. Subsequent molecular studies proposed many new intrafamilial backbone phylogenies of Bombycoidea (e.g., Barber et al. 2015, Breinholt et al. 2017, Kawahara and Barber 2015, Kawahara et al. 2009, Regier et al. 2008a, Zwick 2008, Zwick et al. 2011) and the higher classification of the superfamily has changed significantly, but some parts remain inadequately resolved. At lower levels, there have been only a relatively small number of phylogenetic studies focusing on particular genera (e.g., Ylla et al. 2005, Rubinoff and Le Roux 2008, Kawahara et al. 2013, Ponce et al. 2014, Rubinoff et al. 2017), while new species descriptions continue to accumulate at a very high pace. In particular, in the family Saturniidae nearly 150 species or subspecies have been described per year over the past 10 years on average, thus strongly affecting our current understanding of the diversity of these moths. Other families have received less attention from taxonomists, and can still be considered understudied with many new species awaiting discovery and/or description.

Here, we present a best estimate on the current state of the taxonomic diversity of Bombycoidea, based on the compilation of published nomenclatural acts as well as the consideration of recent phylogenetic work on the superfamily.

**Figure 1.**
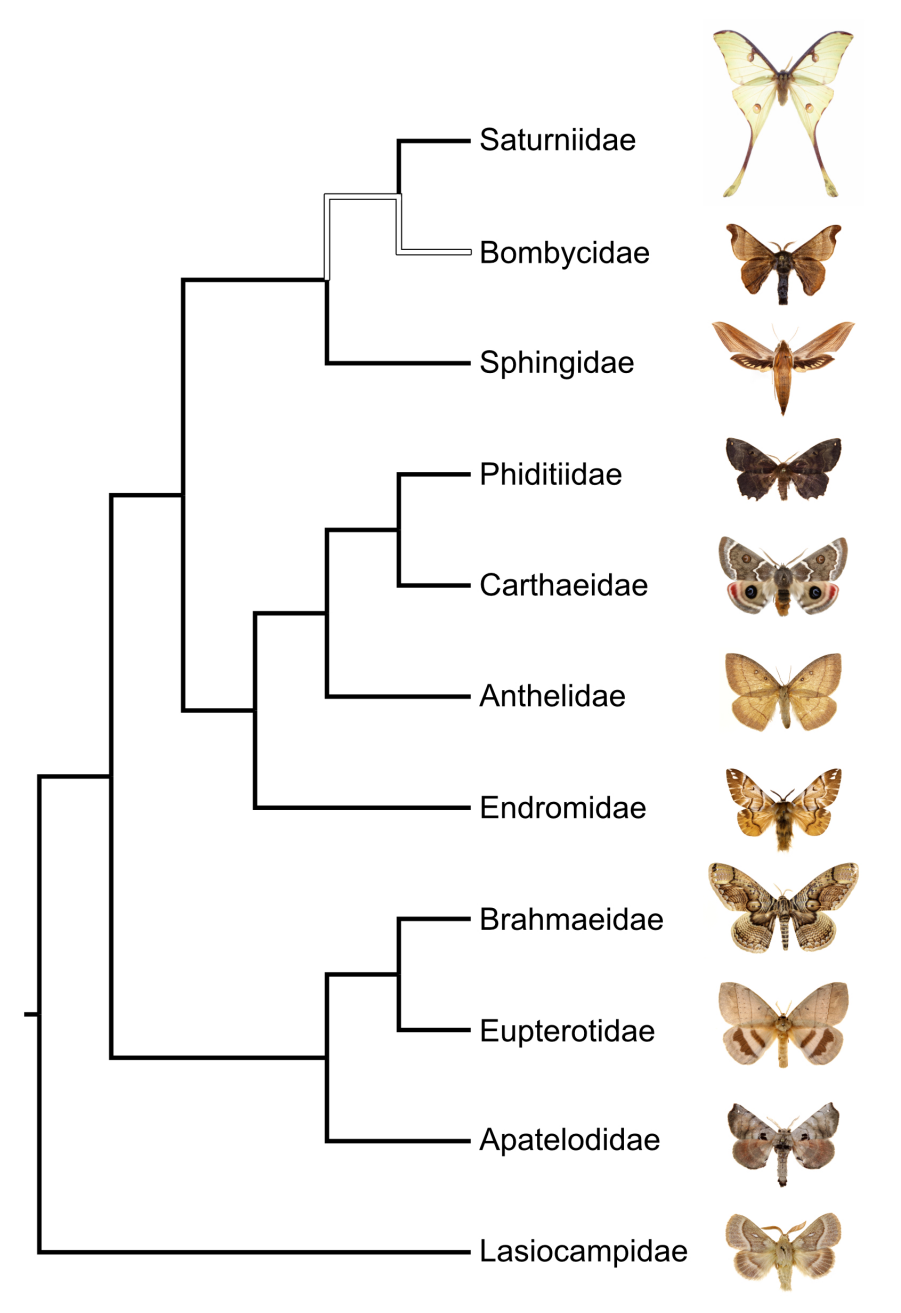
Simplified family-level phylogeny from Zwick et al. (2011). White branch indicates the uncertain placement (i.e., relationship to other families) of the Bombycidae. The closely related family Lasiocampidae is used as an outgroup to root the tree. Photographs at tips are representatives of each family: Saturniidae - *Argema mimosae* (Boisduval, 1847); Bombycidae - *Bombyx mandarina* Moore, 1882; Sphingidae - *Xylophanes tersa* (Linnaeus, 1771); Phiditiidae - *Phiditia* Möschler, 1882 species; Carthaeidae - *Carthaea saturnioides* Walker, 1858; Anthelidae - *Anthela* Walker, 1855 species; Endromidae - *Endromis versicolora* (Linnaeus, 1758); Brahmaeidae - *Brahmaea paukstadtorum* Naumann & Brosch, 2005; Eupterotidae - *Jana eurymas* Herrich-Schäffer, 1854; Apatelodidae - *Apatelodes torrefacta* (Smith, J.E., 1797); Lasiocampidae - *Lasiocampa terreni* (Herrich-Schäffer, 1847).

We have constructed a comprehensive table of bombycoid taxa, including their synonyms, authors, and publication years. Much of this information is erroneous in the literature, and here we comprehensively clarify the taxonomy of the entire superfamily, although we also acknowledge that our checklist may still contain errors and will inevitably become outdated with the expected continued progress in the systematics of these moths. We also present a simplified higher-level phylogeny of Bombycoidea (Fig. 1) based on recent published studies that reflects the taxonomy presented here. Our checklist formally recognize 10 families, 520 genera, and 6,092 species.

## Materials and methods

In this section we provide a list of conventions and abbreviations used, as well as a brief account of the main resources used to compile this checklist for each of the ten families treated.

### Conventions and abbreviations

This Checklist uses the original orthography of all taxon names and does not apply gender agreement (Sommerer 2002).

The following abbreviations and terms are used in the Checklist:

*Code*: the Fourth Edition of the *International Code of Zoological Nomenclature* (1999).

comb. nov.: an new combination of a species into a genus.

comb. rev.: a revived combination of a species into a genus. "Comb. rev." is often misinterpreted as meaning a "revised" combination. However, the term refers specifically to the reinstatement of a previous combination (i.e., a revival, "*reviviscens*"), not a revised combination, which is a more general concept.

incertae sedis: of uncertain taxonomic position.

incorrect original spelling: an original spelling of a name that is deemed incorrect under Articles 32.4 and 32.5 of the *Code*.

infrasubspecific: a name that ranks lower than a subspecies; such names are not regulated by the *Code*.

junior homonym: of two homonyms, the later established, or in the case of simultaneous establishment the one not given precedence under article 24 of the *Code*.

nomen dubium: a name of unknown or doubtful application.

nomen novum (new replacement name): a name expressly established to replace an already established name, most commonly a junior homonym.

nomen nudum: a name that, if published before 1931, fails to conform to Article 12 of the *Code*; or, if published after 1930, fails to conform to Article 13 of the *Code*; an unavailable name, with no type specimen.

nomen oblitum: applied after 1 January 2000 to a name, unused since 1899, which as a result of and action taken under Article 23.9.2 of the *Code* does not take precedence over a younger synonym or homonym in prevailing usage.

nomen protectum: a name that has been given precedence over its unused senior synonym or senior homonym relegated to the status of *nomen oblitum*.

rejected name: a name which, under the provisions of the Code, cannot be used as a valid name and which has been set aside in favour of another name, usually by the application of the plenary powers of the International Commission on Zoological Nomenclature; a name included in a work that has been rejected by the International Commission on Zoological Nomenclature and placed on the *Official Index of Rejected and Invalid Works in Zoological Nomenclature*.

stat. nov.: a new status (e.g., a subspecies name raised to species status for the first time).

stat. rev.: a revived status (e.g., a species name reinstated to species status from synonymy). "Stat. rev." is often misinterpreted as meaning a "revised" status. However, the term refers specifically to the reinstatement of a previous status (i.e., a revival; "*reviviscens*"), not a revised status, which is a more general concept.

syn. nov.: a new synonymy.

syn. rev.: a revived synonymy (i.e the return to synonymic status of a name that had been so treated in the past before being treated as a valid name).

unavailable name: a name that does not conform to Articles 10 to 20 of the *Code*, or that is an excluded name under Article 1.3 of the *Code*.

unnecessary replacement name: a replacement name proposed in error.

unjustified emendation: an intentional change to the original spelling of an available name that is not justified under Article 33.2.2 of the *Code*.

?: of uncertain status.

### Anthelidae Turner, 1904

The classification and nomenclature within Anthelidae follows Edwards and Fairey (1996) for Australian taxa and is based on original descriptions for non-Australian taxa.

### Apatelodidae Neumoegen & Dyar, 1894

The exclusively New World Apatelodidae is treated here as a family (Zwick 2008). The classification and nomenclature follows that of "Apatelodinae" in Becker (1996), with updates from the more recent literature. Of the other two subfamilies included in Apatelodidae by Becker (1996), Epiinae is here treated as a family of Bombycidae and Phiditiinae as a separate family, Phiditiidae, following Zwick et al. (2011).

### Bombycidae Latreille, 1802

Bombycidae is treated here as containing two subfamilies, Bombycinae and Epiinae (Zwick et al. 2011). Of the other subfamilies previously associated with Bombycidae, Apatelodinae and Phiditiinae are treated as families, Phiditiidae and Apatelodidae (Zwick 2008, Zwick et al. 2011), and Oberthueriinae and Prismostictinae are treated as synonyms of Endromidae (Zwick et al. 2011). The classification and nomenclature of Bombycinae follows Beccaloni et al. (2003), and that of Epiinae follows Becker (1996), with updates from the more recent literature.

### Brahmaeidae Swinhoe, 1892

Zwick (2008) found that the lemoniid genera, *Lemonia* Hübner, 1920 and *Sabalia* Walker, 1865, were nested within Brahmaeidae as the sister-group of the African genus *Dactyloceras* Mell, 1930, and to the exclusion of genus *Brahmaea* Walker, 1855, which thus rendered Brahmaeidae paraphyletic. Consequently, he synonymized the two families. Some authors have considered it premature (e.g., Antoshin and Zolotuhin 2013), but Minet (1994) had already recognised the close relationships between the two families on morphological grounds, and all subsequent molecular phylogenetic studies (e.g., Zwick et al. 2011, Regier et al. 2013) have continued to find solid support for Brahmaeidae *sensu* Zwick (2008), and thus it is accepted here. The classification and nomenclature within Brahmaeidae follows Beccaloni et al. (2003), with updates from the more recent literature.

### Carthaeidae Common, 1896

The family Carthaeidae comprises a single genus with a single included species. The classification follows Edwards (1996).

### Endromidae Boisduval, 1828

On the basis of a molecular phylogenetic analysis, Zwick et al. (2011) included the bombycid subfamilies Oberthueriinae and Prismostictinae, and family Mirinidae, within an expanded concept of Endromidae without named subordinate ranks. This re-circumscribed Endromidae so far lacks explicit morphological synapomorphies, and some authors have considered it premature (e.g., Zolotuhin et al. 2011, Zolotuhin 2012, Wang et al. 2015). However, subsequent molecular phylogenetic studies (e.g., Heikkilä et al. 2015) have continued to find good support for Endromidae *sensu Zwick et al. (2011)*, and thus it is accepted here. The generic and species-level classification and nomenclature follows Beccaloni et al. (2003), with updates from the more recent literature.

### Eupterotidae Swinhoe, 1892

The higher classification of Eupterotidae follows Nässig and Oberprieler (2008), and the classification and nomenclature of genera and species follows Beccaloni et al. (2003), with updates from the more recent literature.

### Phiditiidae Minet, 1994

Following Zwick et al. (2011), Phiditiidae is here treated as a family. The classification and nomenclature follows that of "Phiditiinae" in Becker (1996), with updates from the more recent literature.

### Saturniidae Boisduval, 1837

The classification and nomenclature of the New World Saturniidae is based on the revisions of Claude Lemaire (Lemaire 1978, Lemaire 1980, Lemaire 1988, Lemaire 2002), that of the African genera on the checklist by Thierry Bouyer (Bouyer 1999), and that of the remaining taxa on Beccaloni et al. (2003), with updates from the more recent literature.

Nässig et al. (2015) made several adjustments to the higher classification of Saturniidae to reconcile it with the results of several molecular phylogenetic studies (Regier et al. 2002, Regier et al. 2008b, Zwick 2008, Barber et al. 2015). Subfamily Hemileucinae was downgraded to tribal status within subfamily Ceratocampinae, and the saturniine tribe Bunaeini was raised to subfamily status, and tribes Micragonini and Urotini included within it. Although Nässig et al. (2015) appeared to be implementing the principles of phyletic sequencing (Wiley 1979), these changes were poorly justified and represent neither a significant nor a necessary improvement on the current higher classification of Saturniidae. Consequently, pending future comprehensive phylogenetic studies, we here retain the higher classification schemes of Lemaire 1988 and Lemaire 2002 for Ceratocampinae and Hemileucinae, and Oberprieler (1997) for the tribes of Saturniinae.

Based on a subjective, manually constructed cladogram using characters derived mostly from the colour patterns of the adults and larvae, Cooper (2002) proposed new classification, including a number of new tribes, subtribes and genera of African Saturniinae. While we accept that the generic-level classification of tribe Bunaeini (as interpreted here) is highly unsatisfactory (especially that of the *Imbrasia* complex), we consider that the system proposed by Cooper (2002) is superficial and premature (Racheli and Racheli (2006) were of a similar opinion), and should be tested by rigorous phylogenetic methods using both morphological and molecular sequence data before being widely adopted. Consequently, we here synonymize all those taxa newly described by Cooper (2002) (other than those that have already been synonymized by others) pending a more objective analysis of the higher classification of African Saturniinae, and return all the affected species to the genera (and synonymy, if relevant) in which they were previously placed by Bouyer (1999).

### Sphingidae Latreille, 1802

The classification and nomenclature of Sphingidae follows the Sphingidae Taxonomic Inventory (STI) (Kitching 2018). The STI aims to produce a dynamic on-line taxonomic monograph of the Sphingidae within a scratchpad (http://scratchpads.eu) environment, and includes a continually updated taxonomy of the family. Within the STI , each taxon concept ("term") is assigned its own unique URL, underlain by a globally unique identifier (GUID). These URLs and GUIDs are persistent, and do not change regardless of altered taxonomic position in future. For example, the URL for *Sphinx ligustri* Linnaeus, 1758 is http://sphingidae.myspecies.info/taxonomy/term/2632 and the corresponding GUID is 8d338b41-9d48-4378-8af2-5a0ce4c1ceed. In the spreadsheet provided as Suppl. material 1 (Global Bombycoidea checklist), numerous changes to the taxonomy, as currently represented in the printed literature, are noted. Justifications for these taxonomic changes are provided on the corresponding STI taxon pages. Furthermore, the history of taxonomic changes applied to a taxon page is recorded and can be examined by clicking on the "Revisions" tab. To facilitate future studies, and in the interests of open data and transparency, the spreadsheet includes the STI URLs of all taxa for which changes in taxonomic status are here proposed (GUIDs are not given as these can only be seen by registered users with editorial rights). So, for example, we here consider *Ambulyx adhemariusa* Eitschberger, Bergmann & Hauenstein, 2006, to be a junior subjective synonym of *Ambulyx kuangtungensis* (Mell, 1922). The justification for this taxonomic change is given on the STI taxon page for *A. adhemariusa* (http://sphingidae.myspecies.info/taxonomy/term/202). As noted under Data Resources, the cut-off date for inclusion of taxonomic updates (both new taxa and taxonomic changes) in the spreadsheet is 31 January 2018. However, it should be noted that because the STI is a dynamic system, changes will continue to be made as new evidence is forthcoming, so the STI should be consulted for the most up-to-date treatment of any sphingid taxon.

## Data resources

The global checklist of Bombycoidea moths is provided here as a table in Suppl. material 1 (Excel format) providing valid names as well as synonyms for family, genus and species levels. The checklist includes 12,159 names, including synonyms. An account of the number of valid genus and species names per family is given in Table 1. In total, the Bombycoidea superfamily currently comprises 6,092 valid species in 520 valid genera. This checklist is not, however, intended to be a comprehensive revision of the superfamily but represent a "snapshot" of our current taxonomic and nomenclatural knowledge. The cut-off date for inclusion of both new taxa and taxonomic changes was 31 January 2018, but some literature will inevitably have been missed or has yet to be incorporated into the STI. However, the intention is to continue to update the spreadsheet and issue revised versions in the future, whence information on type status, type locality and distribution may be included. Authorships and year of publication are given for all taxa, as well as information regarding the original combination of species with regard to the genus in which they are currently placed in the checklist, as yes (Y), no (N), or currently undetermined ([blank]). For supraspecific taxa, a hyphen (-) is included in this column to indicate "not applicable". Under "Nomenclatural notes", we give details regarding the status of certain names as defined in the Conventions and abbreviations section of Materials and methods (e.g., unjustified emendation). Under "Taxonomic status change", we indicate changes to the current taxonomy to Saturniidae and Sphingidae, as explained in the respective family sections in Materials and methods.

## Checklists

### Bombycoidea checklist

#### Bombycoidea

Latreille, 1802

Bombycoidea See table in Suppl. material 1.

## Supplementary Material

Supplementary material 1 Global Bombycoidea checklist Data type: Taxonomical checklist
Brief description: This table provides a list of 12,159 taxon names for the Bombycoidea superfamily. It includes both valid and synonymous names, with their authorship and information, when known, about the current genus+name binomen being an original combination or not.
File: oo_184138.xlsx Kitching IJK, Rougerie R, Zwick A, St Laurent R, Naumann S

XML Treatment for Bombycoidea

## Figures and Tables

**Table 1.** Number of valid genus and species names in each of the ten families of Bombycoidea.

Family	Number of genera	Number of species
Anthelidae Turner, 1904	9	94
Apatelodidae Neumoegen & Dyar, 1894	12	182
Bombycidae Latreille, 1802	27	202
Brahmaeidae Swinhoe, 1892	6	68
Carthaeidae Common, 1966	1	1
Endromidae Boisduval, 1828	16	70
Eupterotidae Swinhoe, 1892	60	396
Phiditiidae Minet, 1994	4	23
Saturniidae Boisduval, 1837	180	3,454
Sphingidae Latreille, 1802	205	1,602

## References

[B3706113] Antoshin D. A., Zolotuhin V. V. (2013). Taxonomic remarks on the Lemoniidae (Lepidoptera) with description of a new species from Iran. Tinea.

[B3910938] Barber J. R., Kawahara A. Y. (2013). Hawkmoths produce anti-bat ultrasound. Biology Letters.

[B3660699] Barber J. R., Leavell B. C., Keener A. L., Breinholt J. W., Chadwell B. A., McClure C. J.W., Hill G. M., Kawahara A. Y. (2015). Moth tails divert bat attack: evolution of acoustic deflection. Proceedings of the National Academy of Sciences of the United States of America.

[B3699520] Beccaloni G., Scoble M., Kitching I., Simonsen T., Robinson G., Pitkin B., Hine A., Lyal C. The Global Lepidoptera Names Index (LepIndex). http://www.nhm.ac.uk/our-science/data/lepindex/.

[B3698587] Becker V. O., Heppner J. B. (1996). Apatelodidae. Pp. 13-19, 61. Atlas of Neotropical Lepidoptera. Checklist: Part 4B. Drepanoidea - Bombycoidea - Sphingoidea.

[B3701530] Bouyer T. (1999). Catalogue of African Saturniidae. Entomologia Africana, Hors Série.

[B3660713] Breinholt J. W., Earl C., Lemmon A. R., Lemmon E. M., Xiao L., Kawahara A. Y. (2017). Resolving relationships among the megadiverse butterflies and moths with a novel pipeline for anchored phylogenomics. Systematic Biology.

[B3699775] Cooper M. R., Cooper M. R., Cooper M. D. (2002). Note on classification. The emperor moths of KwaZulu-Natal.

[B3698515] Edwards E. D., Nielsen E. S., Edwards E. D., Rangsi T. V. (1996). 80 Carthaeidae. Pp. 623. Monographs on Australian Lepidoptera: Checklist of the Lepidoptera of Australia.

[B3699506] Edwards E. D., Fairey K. D., Neilsen E. S., Edwards E. D., Rangsi T. V. (1996). 77 Anthelidae. Pp. 258-260, 363-364. Monographs on Australian Lepidoptera: Checklist of the Lepidoptera of Australia.

[B3705099] Heikkilä M., Mutanen M., Wahlberg N., Sihvonen P., Kaila L. (2015). Elusive ditrysian phylogeny: an account of combining systematized morphology with molecular data (Lepidoptera). BMC Evolutionary Biology.

[B3660629] Heppner J. B. (1996). Atlas of Neotropical Lepidoptera, Checklist. Part 4B. Drepanoidea, Bombycoidea, Sphingoidea.

[B3660668] Kawahara A. Y., Mignault A. A., Regier J. C., Kitching I. J., Mitter C. (2009). Phylogeny and biogeography of hawkmoths (Lepidoptera: Sphingidae): evidence from five nuclear genes. PLoS ONE.

[B3707008] Kawahara A. Y., Breinholt J. W., Ponce F. V., Haxaire J., Xiao L., Lamarre G. P.A., Rubinoff D., Kitching I. J. (2013). Evolution of *Manduca
sexta* hornworms and relatives: biogeographical analysis reveals an ancestral diversification in Central America.. Molecular Phylogenetics and Evolution.

[B3660679] Kawahara A. Y., Barber J. R. (2015). Tempo and mode of antibat ultrasound production and sonar jamming in the diverse hawkmoth radiation. Proceedings of the National Academy of Sciences of the United States of America.

[B3660620] Kitching I. J., Cadiou J. M. (2000). Hawkmoths of the world: an annotated and illustrated revisionary checklist.

[B3698578] Kitching I. J. Sphingidae Taxonomic Inventory. Creating a Taxonomic e-Science. http://sphingidae.myspecies.info/.

[B3701540] Lemaire C. (1978). Les Attacidae américains. The Attacidae of America (= Saturniidae). Attacinae.

[B3701549] Lemaire C. (1980). Les Attacidae américains. The Attacidae of America (= Saturniidae). Arsenurinae.

[B3701558] Lemaire C. (1988). Les Saturniidae américains. The Saturniidae of America. Los Saturniidae americanos (= Attacidae) Ceratocampinae.

[B3702341] Lemaire C. (2002). The Saturniidae of America. Les Saturniidae américains. The Saturniidae of America (= Attacidae) Hemileucinae.

[B3810479] Minet Joel (1991). Tentative reconstruction of the ditrysian phylogeny (Lepidoptera: Glossata). Insect Systematics & Evolution.

[B3810489] Minet Joel (1994). The Bombycoidea: Phylogeny and higher classification (Lepidoptera: Glossata). Insect Systematics & Evolution.

[B3706080] Nässig W. A., Oberprieler R. G. (2008). An annotated catalogue of the genera of Eupterotidae (Insecta, Lepidoptera, Bombycoidea). Senckenbergiana Biologica.

[B3705720] Nässig W. A., Naumann S., Oberprieler R. G. (2015). Notes on the Saturniidae of the Arabian Peninsula, with description of a new species (Lepidoptera: Saturniidae). Nachrichten des Entomologischen Vereins Apollo (N.F.).

[B3706060] Oberprieler R. G. (1997). Classification of the African Saturniidae (Lepidoptera) - the quest for natural groups and relationships. Metamorphosis Occasional Supplement.

[B3707054] Ponce F. V., Breinholt J. W., Hossie T., Barber J. R., Janzen D. H., Hallwachs W., Kawahara A. Y. (2014). A molecular phylogeny of *Eumorpha* (Lepidoptera: Sphingidae) and the evolution of anti-predator larval eyespots. Systematic Entomology.

[B3705949] Racheli L., Racheli T. (2006). Phylogenetic hypothesis and classification: theoretical and methodological issues with reference to some studies on Saturniidae (Lepidoptera: Saturniidae). SHILAP Revista de Lepidopterología.

[B3705730] Regier J. C., Friedlander T. P., Mitter C., Peigler R. S. (2002). Monophyly, composition, and relationships within Saturniinae (Lepidoptera: Saturniidae): evidence from two nuclear genes. Insect Systematics & Evolution.

[B3660689] Regier J. C., Cook C. P., Mitter C., Hussey A. (2008). A phylogenetic study of the 'bombycoid complex' (Lepidoptera) using five protein-coding nuclear genes, with comments on the problem of macrolepidopteran phylogeny. Systematic Entomology.

[B3705755] Regier J. C., Grant M. C., Mitter C., Cook C. P., Peigler R. S., Rougerie R. (2008). Phylogenetic relationships of wild silkmoths (Lepidoptera: Saturniidae) inferred from four protein-coding nuclear genes. Systematic Entomology.

[B3705690] Regier J. C., Mitter C., Zwick A., Bazinet A. L., Cummings M. P., Kawahara A. Y., Sohn J. C., Zwickl D. J., Cho S., Davis D. R., Baixeras J., Brown J., Parr C., Weller S., Lees D. C., Mitter K. T. (2013). A large-scale, higher-level, molecular phylogenetic study of the insect order Lepidoptera (moths and butterflies). PLoS ONE.

[B3706946] Roe A. D., Weller S. J., Baixeras J., Brown J., Cummings M. P., Davis D., Kawahara A. Y., Parr C., Regier J. C., Rubinoff D., Goldsmith M., Marec F. (2009). Evolutionary framework for Lepidoptera model systems, pp. 1-24. Genetics and Molecular Biology of Lepidoptera.

[B3706998] Rubinoff D., Le Roux J. J. (2008). Evidence of repeated and independent saltational evolution in a peculiar genus of sphinx moths (*Proserpinus*: Sphingidae). PLoS ONE.

[B3660735] Rubinoff D., San Jose M., Peigler R. S. (2017). Multi-gene phylogeny of the *Hemileuca
maia* complex (Saturniidae) across North America suggests complex phylogeography and rapid ecological diversification. Systematic Entomology.

[B3699467] Sommerer M. D. (2002). To agree or not to agree - the question of gender agreement in the International Code of Zoological Nomenclature. Nota Lepidopterologica.

[B3660562] van Nieukerken E. J., Kaila L., Kitching I. J., Kristensen N. P., Lees D. C., Minet J., Mitter C., Mutanen M., Regier J. C., Simonsen T. J., Wahlberg N., Yen S. H., Zahiri R., Adamski D., Baixeras J., Bartsch D., Bengtsson B. Å., Brown J. W., Bucheli S. R., Davis D. R., De Prins J., De Prins W., Epstein M. E., Gentili-Poole P., Gielis C., Hättenschwiler P., Hausmann A., Holloway J. D., Kallies A., Karsholt O., Kawahara A. Y., Koster S., Kozlov M. V., Lafontaine J. D., Lamas G., Landry J. F., Lee S., Nuss M., Park K. T., Penz C., Rota J., Schmidt B. C., Schintlmeister A., Sohn J. C., Solis M. A., Tarmann G. M., Warren A. D., Weller S., Yakovlev R. V., Zolotuhin V. V., Zwick A. (2011). Order Lepidoptera Linnaeus, 1758. Zootaxa.

[B3703298] Wang Xi, Wang M., Zolotuhin V. V., Hirowatari T., Wu S., Huang G. H. (2015). The fauna of the family Bombycidae sensu lato (Insecta, Lepidoptera, Bombycoidea) from Mainland China, Taiwan and Hainan Islands.. Zootaxa.

[B3705786] Wiley E. O. (1979). An annotated Linnaean hierarchy, with comments on natural taxa and competing systems. Systematic Zoology.

[B3660725] Ylla J., Peigler R. S., Kawahara A. Y. (2005). Cladistic analysis of moon moths using morphology, molecules and behaviour: *Actias* Leach, 1815; *Argema* Wallengren, 1858; *Graellsia* Grote, 1896 (Lepidoptera: Saturniidae). SHILAP Revista Lepidopterologíca.

[B3705110] Zolotuhin V. V., Pugaev S. N., Sinjaev V. V., Witt T. J. (2011). The biology of Mirinidae with description of preimaginal instars of *Mirina
confucius* Zolotuhin & Witt, 2000 (Lepidoptera, Mirinidae). Tinea.

[B3703255] Zolotuhin V. V. (2012). Taxonomic remarks on *Andraca* Walker, 1865 (Lepidoptera: Bombycidae) with descriptions of five new species. Zootaxa.

[B3660648] Zwick A. (2008). Molecular phylogeny of Anthelidae and other bombycoid taxa (Lepidoptera: Bombycoidea). Systematic Entomology.

[B3660658] Zwick A, Regier J. C, Mitter C, Cummings M. P (2011). Increased gene sampling yields robust support for higher-level clades within Bombycoidea (Lepidoptera). Systematic Entomology.

